# Association of duration of undernutrition with occurrence of tuberculosis

**DOI:** 10.1186/s12889-022-14876-1

**Published:** 2022-12-20

**Authors:** Jiho Park, Ji Hyun Yoon, Hyun Kyun Ki, Yeonghee Eun, Kyungdo Han, Hyungjin Kim

**Affiliations:** 1 Department of Medicine, Konkuk University of Medical Center, Konkuk University School of Medicine, 05030 Seoul, South Korea; 2 Department of Medicine, Samsung Medical Center, Sungkyunkwan University School of Medicine, 06351 Seoul, South Korea; 3 Department of Statistics and Actuarial Science, Soongsil University, 369, Sangdo-ro, Dongjak-gu, 06978 Seoul, South Korea; 4 Department of Medical Humanities, Samsung Medical Center, Sungkyunkwan University School of Medicine, 81 Irwon-ro, Gangnam-gu, 06351 Seoul, South Korea

**Keywords:** Underweight, Tuberculosis, Nationwide data

## Abstract

Undernutrition is a risk factor of tuberculosis (TB), but the association between duration of undernutrition and occurrence of TB is inconclusive. The objective of this study was to determine whether there is a difference in occurrence of TB according to the duration of undernutrition expressed as accumulated number of underweight in Republic of Korea, an intermediate TB burden country. The National Health Insurance database was used.

Eligible subjects were individuals who received a national health examination between 1 and 2009 and 31 December 2010, and who also had received health examinations for four consecutive years prior to 2009.

Finally included individuals in the analysis were followed until 31 December 2017. Accumulated number of underweight was defined as the number of times recorded as underweight over four consecutive years. The outcome of the study was newly diagnosed TB according to accumulated number of underweight. Among a study population of 2,396,434, TB was identified in 9,322 (3.89%) cases. The highest accumulated number of underweight was significantly associated with occurrence of TB (adjusted hazard ratio [aHR] 2.563, 95% CI 2.319–2.833). This association remained consistent after adjusting for demographic factors and underlying diseases (aHR 3.326, 95% CI 3.004–3.84). In stratified analysis based on age, sex, diabetes (DM), hypertension (HTN), and waist circumference (WC) in metabolic syndrome (MS), age and sex were identified as effect modifiers. Occurrence of TB was significantly higher in the group with the highest accumulated number of underweight under 65 years of age.

## Introduction

The World Health Organization (WHO) reported about 10.58 million new and relapse TB cases globally and approximately 1.53 million TB-related deaths including 214,000 people with HIV worldwide in the year 2020 [1]. TB control and eradication depend on early detection of active TB patients, prompt anti-TB treatment, identification of persons in risk of TB exposure and prevention of secondary TB cases. All these management rely on not only development of diagnostic method but also social determinants, for example, social and risk behaviors, environment, income, health care cost and nutrition. Taking these factors into account, vigorous national measures for controlling TB have been implemented over the decades, and the incidence and death rates of TB were declining yearly. However, deaths from tuberculosis remain high in low-income countries [1, 2]. TB began to be recognized as a health problem in the Republic of Korea (hereafter referred to as “Korea”) from 1950s. After the end of the Korean War in 1953, Korea had achieved remarkable success in economic growth with significant improvements in quality of life over the past decades. However, the increase of TB burden in Korea is unresolved despite long-term strategic planning of the national TB control program accompanying national development. The number of new TB cases in Korea in 2020 was 19,933 persons, a decrease of 16.3% from the previous year (23,821 persons). Since 2010, it achieved an average annual decrease of 5.8% [3]. Nevertheless, it still has the highest TB incidence rate (49 per 100,000 population) among the 38 member countries of the Organization for Economic Cooperation and Development (OECD) and third highest rate of mortality from TB (38 per 100,000 population) [3].

There are several established risk factors for TB. Underweight is one of the risk factor for development of TB [4–10]. Undernutrition is associated with progression from latent TB to active TB likely due to impairment of the cell-mediated immune system [6–8]. A more than three-fold increased risk of TB was noted in people with underweight compared to those with normal body weight [8]. However, these studies had focused on an association between underweight at one time point and occurrence of TB. The association between accumulated number of underweight for several years and occurrence of TB has not been studied. The aim of the present study was to determine whether there is a difference in occurrence of TB according to accumulated number of underweight based on data from national health examinations in Korea, which is as an intermediate TB burden country.

## Methods

### Data source and study populations

In Korea, the Korean National Health Insurance Service (NHIS), a single insurer managed by the Korean government, provides a mandatory universal health insurance to 97% of the population. The NHIS provides free biennial national health examination programs, which include a general health examination for all citizens aged 40 and above and all employees older than 20 years.

These exams include anthropometric measurements (i.e., height, weight, and waist circumference [WC]); blood pressure (BP); laboratory measurements; and lifestyle information. The NHIS also keeps medical data, including diagnostic codes, procedures and operations, prescription drugs, and intractable diseases including cancer.

Our study used the health examination data and medical data from the NHIS. We included the data of people who received a health examination through the NHIS between 1 and 2009 and 31 December 2010, and who had received health examinations for four consecutive years prior to 2009. The observation period of change of body weight was from 2006 to 2009, and subjects diagnosed with TB during this period were excluded considering that tuberculosis is a slowly progressing chronic infectious disease, it was assumed that the effect of weight change would not appear immediately within same observational period. We also excluded individuals with any missing variables, and those who were diagnosed with TB or died within one year after the national health examination. Included populations in the analysis were followed until 31 December 2017.

The present study was approved by the Institutional Review Board (IRB) of Konkuk university of medical center (#2022-03-028). The study was performed in accordance with the guidelines followed as per the Declaration of Helsinki. Informed consent was waived by the IRB of Konkuk university of medical center.

### Measurement and definitions

During the national health examination, height, weight, and WC were measured. Body mass index (BMI) was calculated by dividing weight (kg) by height (m) squared and was classified as underweight (< 18.5 kg/m2), normal to overweight (18.5–24.9 kg/m2), or obese (≥ 25 kg/m2) by WHO [11, 12]. Accumulated number of underweight was defined as the number of times recorded as underweight at the national health examination during the four consecutive years prior to the health examination in 2009–2010. We investigated the accumulated number of underweight of the included population. Study populations responded to a standardized self-administered questionnaire regarding past medical history and lifestyle behaviors such as smoking, and drinking. Smoking status was classified into never smoker, ex-smoker, and current smoker. Drinking was divided into none, mild (< 30 g of alcohol/day), and heavy (≥ 30 g/day) drinking.

Baseline co-morbidities included hypertension (HTN), diabetes mellitus (DM), and dyslipidemia. These diseases were defined using physician diagnosis or use of medication based on self-reporting. HTN was defined as systolic blood pressure (BP) ≥ 140 mmHg, diastolic BP ≥ 90 mmHg, or use of antihypertensive drugs with a prior diagnosis (International Classification of Diseases, Tenth Revision, Clinical Modification [ICD-10-CM] codes I10-13, I15). BP was measured after the subjects had been seated for 5 min with the arm in the appropriate position. Similarly, DM was defined as a prior diagnosis (ICD-10-CM codes E11-14) and treatment with glucose-lowering agents or as overnight fasting plasma glucose ≥ 126 mg/dL. Dyslipidemia was defined as a prior diagnosis (ICD-10-CM code E78) and treatment with statins or as total cholesterol ≥ 240 mg/dL. Definition of WC in metabolic syndrome (MS) which reflects abdominal obesity was that WC ≥ 90 cm for men, or ≥ 85 cm for women [13, 14]. Whether TB occurred or not after 2010 was followed up for 7.27 years. Those who developed TB within one year of health examination and those who died in any reason within one year of health examination were excluded.

### Study outcomes and follow-up

The outcome of the study was occurrence of newly diagnosed TB according to accumulated number of underweight. The NHIS provided additional insurance coverage for all patients diagnosed with cancer and some rare diseases such as TB to enhance benefit coverage from the year 2010. Specific insurance codes were applied mandatorily to patients with TB after confirmation of diagnosis [15, 16]. The cohort was followed from 1 year after the health examination date to the date of occurrence of TB or until the end of the study period.

### Statistical analysis

To compare clinical variables, the kruskal wallis test was used for continuous variables and the χ2 test for categorical variables. The event rate was estimated using the Kaplan–Meier method, and between-group comparisons of the resulting curves were subjected to a univariate analysis via the log-rank test. Cox proportional hazards analyses were performed to evaluate the association of accumulated number of underweight with occurrence of TB, and the associations were calculated as hazard ratio (HR) and 95% confidence interval (CI). A multivariable-adjusted proportional hazards model was applied: (1) Model 1 was unadjusted; (2) Model 2 was adjusted for age, sex, DM, HTN, and dyslipidemia. In addition, stratified analysis according to sex, age, presence of DM, presence of HTN, and WC in MS was performed because these factors have a significant impact on TB [17, 18]. Statistical analyses were conducted using SAS software (Version 9.4; SAS Institute, Cary, NC, USA), and a *P*-value < 0.05 was considered statistically significant.

## Results

### Baseline characteristics of study populations

**Fig. 1:**
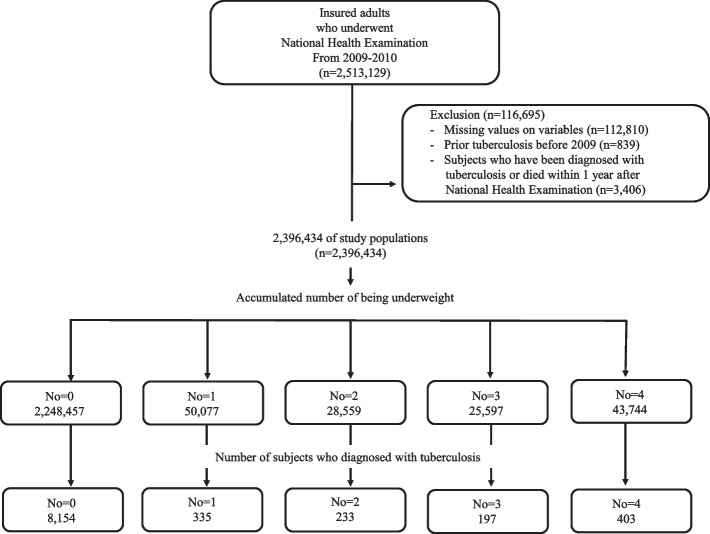
Study population

Of the 2,513,129 people who had undergone health examinations for four consecutive years prior to 2009, 2,396,434 eligible subjects were included in the analysis after exclusion. During a median follow-up of 7.27 years after a 1-year lag period, 9,322 (3.59%) people were diagnosed with TB (Fig. 1). Baseline characteristics of the study population according to accumulated number of underweight are described in Table 1. There were 2,248,457 (93.8%) subjects with zero accumulated number of underweight, 50,077 (2.08%) subjects with one accumulated number of underweight, 28,559 (1.19%) subjects with two accumulated number of underweight, 25,597 (1.06%) subjects with three accumulated number of underweight, and 43,744 (1.82%) subjects with four accumulated number of underweight. Those with a higher accumulated number of underweight appeared to be younger, more typically female, non-smokers, and non-drinkers. They also appeared to have fewer DM, HTN and dyslipidemia (Table 1).

**Table 1:** Baseline characteristic of the study populations according to the accumulated number of underweight

	Accumulated number of being underweight					
	0	1	2	3	4	*p* value
	2,248,457 (93.8)	50,077 (2.08)	28,559 (1.19)	25,597 (1.06)	43,744 (1.82)	< 0.0001
**Age**	43 (34 − 5)	34 (29–45)	32 (28–43)	32 (28–39)	32 (28–39)	< 0.0001
20–39	1,016,130 (45.19)	33,524 (66.94)	20,523 (71.86)	19,236 (75.15)	33,418 (76.39)	
40–64	1,188,218 (52.85)	15,798 (31.55)	7663 (26.83)	6049 (23.63)	9803 (22.41)	
**65≥**	44,109 (1.96)	755 (1.51)	373 (1.31)	312 (1.22)	523 (1.2)	
**Sex**						< 0.0001
Male	1,722,635 (76.61)	23,537 (47)	12,016 (42.07)	10,199 (39.84)	16,720 (38.22)	
Female	525,822 (23.39)	26,540 (53)	16,543 (57.93)	15,398 (60.16)	27,024 (61.78)	
**Smoking**						< 0.0001
Non	986,840 (43.89)	30,893 (61.69)	18,459 (64.63)	16,888 (65.98)	29,400 (67.21)	
Ex	463,362 (20.61)	5134 (10.25)	2611 (9.14)	1964 (7.67)	3040 (6.95)	
Current	798,255 (35.5)	14,050 (28.06)	7489 (26.22)	6745 (26.35)	11,304 (25.84)	
**Drink**						< 0.0001
Non	846,399 (37.64)	24,188 (48.3)	14,259 (49.93)	13,031 (50.91)	22,566 (51.59)	
Mild	1,214,930 (54.03)	23,561 (47.05)	13,157 (46.07)	11,528 (45.04)	19,637 (44.89)	
Heavy	187,128 (8.32)	2328 (4.65)	1143 (4)	1038 (4.06)	1541 (3.52)	
**DM**	164,746 (7.33)	1588 (3.17)	745 (2.61)	590 (2.3)	836 (1.91)	< 0.0001
**HTN**	448,078 (19.93)	3389 (6.77)	1500 (5.25)	1169 (4.57)	1735 (3.97)	< 0.0001
**Dyslipidemia**	363,881 (16.18)	3155 (6.3)	1526 (5.34)	1357 (5.3)	1549 (3.54)	< 0.0001
**WC**	81 (76–87)	69 (66–74)	68 (64–72)	67 (63–71)	65 (62–69)	< 0.0001
**BMI**	23.8 (21,94-25.78)	19.05 (18.51–19.77)	18.59 (18.18–19.1)	18.25 (17.78–18.67)	17.47 (16.9-17.94)	< 0.0001

Data are expressed as number (%) of subjects or median (IQR)

*DM* Diabetes mellitus, *HTN* Hypertension, *WC* Waist circumference, *BMI* Body mass index

### Associations of accumulated number of underweight with occurrence of TB

**Fig. 2:**
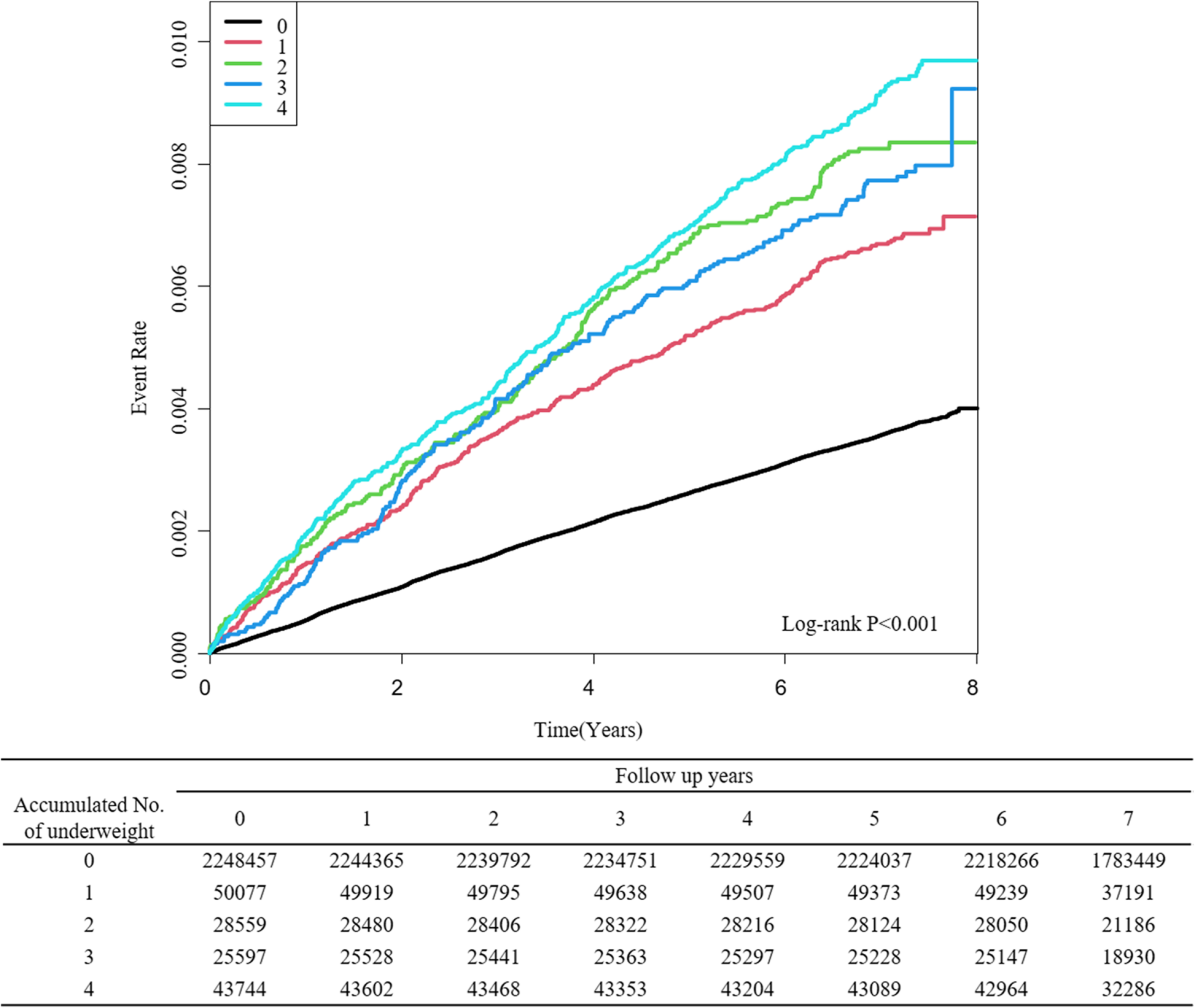
Kaplan–Meier estimation of the event rate of TB. The analyses are stratified by accumulated number of being underweight

The highest accumulated number of underweight was significantly associated with occurrence of TB. In patient with the highest accumulated number of underweight demonstrated the highest risk of occurrence of TB (aHR 2.563, 95% CI 2.319–2.833) (Table 2).This association remained consistent after adjusting for demographic factors and underlying diseases (aHR 3.326, 95% CI 3.004–3.84) (Table 2). The Kaplan–Meier estimations of the event rates according to accumulated number of underweight are shown in Fig. 2. Notably, a higher event rate was observed at higher accumulated number of underweight.

**Table 2:** Hazard ratios (HR) and 95% confidence intervals (CI) of occurrence of TB by accumulated number of being underweight

	N.	TB	Follow-up Duration, Person-year	IR per 1000	Model 1^a^, HR (95% CI)	*p*-value	Model 2^b^, HR (95% CI)	*p*-value
Accumulated number of underweight								
0	2,248,457	8154	16047688.45	0.50811	1(Ref.)	< 0.0001	1(Ref.)	< 0.0001
1	50,077	335	354086.38	0.9461	1.86 (1.668–2.075)		2.208 (1.978–2.465)	
2	28,559	233	201932.53	1.15385	2.269 (1.992–2.584)		2.84 (2.49–3.238)	
3	25,597	197	180870.79	1.08918	2.141 (1.859–2.467)		2.743 (2.379–3.164)	
4	43,744	403	309112.94	1.30373	2.563 (2.319–2.833)		3.326 (3.004–3.684)	

*TB* Tuberculosis, *IR* Incidence rate, *HR* Hazard ratio, *CI* Confidence interval

^a^Model 1: Non-adjusted

^b^Model 2: adjusted for age, sex, diabetes mellitus, hypertension and dyslipidemia

### Stratified analysis of the covariates on the association between BMI or accumulated number of underweight and occurrence of tuberculosis

The potential impact of other independent factors on the association of accumulated number of underweight with occurrence of TB was evaluated by stratified analysis. When we performed stratified analysis according to age, sex, DM, HTN, and WC in metabolic syndrome (MS), only age and sex were effect modifiers. The association between accumulated number of underweight has an increasing trend for young age group and female, but not for age 40–64, over 65, or male (Table 3).

**Table 3:** The association of accumulated number of being underweight with occurrence of tuberculosis stratified by various independent factors

	**Accumulated number of underweight**	**N.**	**TB**	**Duration**	**IR per 1000**	**Estimated HR** **(95% CI)**	***p*-value**	***p* for interaction**
**Age**								
20-39	0	1016130	2784	7254828.86	0.38374	1(Ref.)	<0.0001	0.0081
	1	33524	162	237713.29	0.68149	1.694 (1.441-1.991)		
	2	20523	117	145599.57	0.80357	1.987 (1.645-2.4)		
	3	19236	124	136333.74	0.90953	2.244 (1.867-2.698)		
	4	33418	225	236914.54	0.94971	2.335 (2.026-2.692)		
40-64	0	1188218	4893	8494372.34	0.57603	1(Ref.)	<0.0001	
	1	15798	147	111500.76	1.31838	2.258 (1.915-2.661)		
	2	7663	101	53923.95	1.87301	3.235 (2.655-3.942)		
	3	6049	63	42535.48	1.48112	2.489 (1.94-3.193)		
	4	9803	160	68873.4	2.3231	3.931(3.355-4.606)		
**65≤**	0	44109	477	298487.25	1.59806	1(Ref.)	<0.0001	
	1	755	26	4872.33	5.33626	3.054 (2.054,4.543)		
	2	373	15	2409.01	6.22661	3.494 (2.084,5.859)		
	3	312	10	2001.57	4.99607	2.862 (1.527,5.366)		
	4	523	18	3325	5.41354	2.962 (1.841,4.764)		
**Sex**								
Male	0	1722635	6330	12313526.09	0.51407	1(Ref.)	<0.0001	<0.0001
	1	23537	214	165917.89	1.28979	2.534 (2.211-2.905)		
	2	12016	151	84597.6	1.78492	3.568 (3.035-4.195)		
	3	10199	111	71655.97	1.54907	3.08 (2.551-3.717)		
	4	16720	219	117699.64	1.86067	3.694 (3.226-4.23)		
Female	0	525822	1824	3734162.36	0.48846	1(Ref.)	<0.0001	
	1	26540	121	188168.49	0.64304	1.464 (1.214-1.765)		
	2	16543	82	117334.93	0.69885	1.624 (1.297-2.034)		
	3	15398	86	109214.82	0.78744	1.852 (1.486-2.31)		
	4	27024	184	191413.3	0.96127	2.271 (1.939-2.66)		
**DM**								
No	0	2083711	7009	14887011.91	0.47081	1(Ref.)	<0.0001	0.084
	1	48489	319	343191.48	0.92951	2.245 (2.004-2.514)		
	2	27814	213	196854.41	1.08202	2.726 (2.376-3.128)		
	3	25007	190	176838.16	1.07443	2.768 (2.393-3.202)		
	4	42908	381	303475.62	1.25546	3.252 (2.928-3.613)		
Yes	0	164746	1145	1160676.55	0.98649	1(Ref.)	<0.0001	
	1	1588	16	10894.9	1.46858	1.477 (0.901-2.422)		
	2	745	20	5078.12	3.93846	4.237 (2.719-6.603)		
	3	590	7	4032.62	1.73584	1.807 (0.859-3.802)		
	4	836	22	5637.32	3.90256	4.071(2.664-6.22)		
**HTN **								
No	0	1800379	6203	12869752.79	0.48198	1(Ref.)	<0.0001	0.0599
	1	46688	298	330517.49	0.90162	2.129 (1.894-2.395)		
	2	27059	209	191559.24	1.09105	2.68 (2.332-3.08)		
	3	24428	186	172786.48	1.07647	2.699 (2.329-3.127)		
	4	42009	366	297239.81	1.23133	3.105 (2.788-3.458)		
Yes	0	448078	1951	3177935.66	0.61392	1(Ref.)	<0.0001	
	1	3389	37	23568.89	1.56987	2.386 (1.723-3.305)		
	2	1500	24	10373.29	2.31363	3.667 (2.45-5.489)		
	3	1169	11	8084.3	1.36066	2.073 (1.145-3.751)		
	4	1735	37	11873.13	3.11628	4.932 (3.558-6.838)		
**WC in MS**								
No	0	1860853	7111	13289090.35	0.5351	1(Ref.)	<0.0001	0.4696
	1	49608	334	350754.51	0.95223	2.08 (1.862-2.323)		
	2	28412	232	200883.64	1.1549	2.656 (2.328-3.03)		
	3	25415	196	179573.13	1.09148	2.565 (2.223-2.959)		
	4	43713	402	308893.74	1.30142	3.111(2.808-3.446)		
Yes	0	387604	1043	2758598.1	0.37809	1(Ref.)	0.0105	
	1	469	1	3331.87	0.30013	0.999 (0.141-7.104)		
	2	147	1	1048.9	0.95338	5.246 (0.735-37.426)		
	3	182	1	1297.66	0.77062	5.286 (0.738-37.854)		
	4	31	1	219.2	4.5621	16.219 (2.289-114.917)		

*TB* Tuberculosis, *IR* Incidence rate, *HR* Hazard ratio, *CI* Confidence interval, *DM* Diabetes mellitus, *HTN* Hypertension, *WC* Waist circumference, *MS* Metabolic syndrome

## Discussion

In this large-scale, population-based, longitudinal study, we investigated the association between accumulated number of underweight and occurrence of TB, and then we found that longer duration of underweight with had a higher association with occurrence of TB. According to a detailed analysis of the study results, we found that subjects with a higher accumulated number of underweight appeared to be younger, more typically female, non-smokers, and non-drinkers.

This appears to be a result of the fact that Korean women are overwhelmingly underweight compared to men, and there are more non-smokers, and non-drinkers than men. It is known that approximately two to three times more women than men who do not smoke or drink at all, and ten times more men than women who smoke and drink in Korea [19]. It is believed that the reason why many Korean women are underweight is the difference perception between men and women about their ideal body shape. Young unmarried women might expect themselves to be attractive to potential partners by obtaining their ideal body shape and women tend to perceive a leaner body shape as ideal compared to men who want to have a muscular body shape. This difference in ideal body shape between men and women may be an important cause explaining the finding that young women was related to being underweight in our study [20].

Those with a higher accumulated number of underweight also had a higher association with occurrence of TB [19]. Underweight has been shown to be associated with host susceptibility to TB in several studies. In a similar context, several studies have shown overweight people to have a lower incidence of TB [4–10]. Lee et al. explored the prevalence rate of active TB among homeless people who had poor nutrition and unsafe housing conditions in Seoul, Korea. In that study, underweight, defined as BMI < 18.5, was an independent risk factor for active pulmonary TB [4]. Kim et al. evaluated the association between BMI and incidence of TB and showed that incident TB decreased as BMI increased after adjusting for age, sex, income, smoking, alcohol, and diabetes [10]. Badawi et al. performed systematic analysis of the relationship between obesity and TB and reported that the adjusted odds ratio of TB was 4.96 in underweight people and 0.26 in obese people [8]. Another systematic literature review found a strong and consistent log-linear inverse relationship between BMI and TB incidence in countries with a variety of TB burdens [6]. These studies were designed so that the subjects’ body weight was assessed at a certain point in time, and that those subjects were followed for several years to observe whether TB develops. There are no data on whether a prolonged previous underweight affects occurrence of TB.

However, monitoring of weight history is important in health outcomes. Weight fluctuation or weight cycling can indicate difficulty maintaining homeostasis and reflect changes in body composition such as fat mass or lean mass. These changes in weight might be associated with increased risk of all-cause mortality, especially in older adults [21–23].

Based on these studies, we introduced the definition of accumulated number of underweight and investigated whether occurrence of TB increased as accumulated number of underweight increased. Our study found that occurrence of TB was significantly higher among those who had been underweight for all 4 years, especially age under 65. Occurrence of TB in Korea increases with age and is most prevalent in the elderly, especially in those aged 65 years or over [24]. Many physiological changes of lung function are associated with aging such as a decrease in the elastic recoil of the lung and compliance of the chest wall. Sarcopenia associated with respiratory muscle dysfunction is also common in the elderly [24, 25]. In people over 65 years of age, it seems that underweight is not identified as the effect modifier of TB due to this large effect of the age-related decline in lung function.

Underweight could be an indicator for undernutrition and a surrogate marker for frailty. It is well known that undernutrition is linked closely to cellular immune function. Nutrition regulates changes in immune cells, which alter immunity in terms of infection response in human studies [7, 26–28]. The results of experiments aiming to identify the underlying mechanism of these changes point to defects in T cell recirculation and proliferation and diminished production of protective cytokines and anti-mycobacterial effector molecules [7, 29, 30]. Nevertheless, the concept of underweight is a heterogenous and complex condition found in otherwise healthy subjects as well as patients with underlying disease, several studies suggested that underweight are associated with increased infection risk [31].

The strength of our study is that it is a nationwide, population-based study including a large study population of more than two million. Because of the high participation rate of national health examinations, there is a small possibility of selection bias, and the results of this study have high generalizability. In addition, the detailed personal data collected at baseline enabled us to stratify and adjust for relevant risk factors for TB. Furthermore, there was 1 year of time lag between the latest record of weight calculation and TB diagnosis based on ICD codes. In general, retrospective cohort studies have a limitation of not being able to determine cause-effect relationships, but by setting a time lag, the cause-effect relationship could be explained even in a retrospective study design. Moreover, because our study collected body weight over four years, we could evaluate the time-varying effect of underweight on occurrence of TB.

Although TB is significantly associated with malnutrition, especially in poorer countries or war-time conditions, this type of malnutrition does not occur or occurs in a very small fraction of the population in Korea [7]. Mild to moderate undernutrition can affect large fractions of the population at risk for TB in Korea. This risk relative to specific levels of undernutrition should be defined. Our study presented the concept of accumulated number of underweight and indirectly suggested the severity of the undernutrition condition through these numbers. Latent tuberculosis infection (LTBI) is considered a critical factor for high TB prevalence in Korea. Therefore, programmatic management of LTBI is one of the key strategies of national TB control programs, and LTBI management focusing on this strategy has expanded every year. The most important strategy of LTBI management is prioritization of the target population, those who were at high risk of developing TB [32, 33]. Based on the results of this study, it is expected that LTBI treatment could be considered in those who have maintained underweight for several years.

There are several limitations to our study. First, the universal validity of our results might be in question because almost all of our study subjects were Korean. The generalizability of our findings to other non-Asian ethnic groups requires further investigation. Second, we had no information about the effect of body composition. Lean mass and fat mass would have different effects on development of TB [34]. However, underweight does not distinguish between adipose fat, muscle, bone, and water. To overcome this limitation, we performed stratified analysis according to WC and MS to minimize the effect of adiposity. Third, since an experimental study was not carried out, we could not elucidate the exact pathophysiology of the effect of duration of underweight on occurrence of TB. More research is needed to clarify the pathophysiology, focusing on TB and impairment of immune function according to accumulated number of underweight. Fourth, we assumed that each underweight event happened in any year during the 4 years prior to 2009 was same. If a person has a BMI close to the cut off, he or she is more likely to have random nutritional status switch during the four years. In this case, accumulated number of underweight may not much relevant to the duration of malnutrition. Furthermore, accumulated number of underweight is not the number of consecutively confirmed underweight. However, it can be considered that there is an effect with duration of underweight because the occurrence of TB significantly increased in subjects who were underweight over 4 years, with the accumulated number of times of underweight being 4 times. Fifth, we did not analyze severe underweight with a BMI of less than 16.5 kg/m2. If this analysis is done separately in the future, it is expected that it will be more helpful in policy making. We also did analyze subjects with human immunodeficiency virus (HIV), which commonly causes undernutrition, because korea has a very low prevalence of HIV. Finally, We excluded subjects diagnosed with TB from 2006 to 2009 considering that tuberculosis is a slowly progressing chronic infectious disease, however, we overlooked the effect of nutritional shocks which can become apparent in populations within months through strong historical evidence, such as nutritional emergency in Venezuela [35].

In conclusion, accumulated number of underweight had a statistically significant association with occurrence of TB even after adjusting for various independent factors.

## Data Availability

The datasets analyzed during the current study are available from the corresponding author on reasonable request.
